# Optimal blood pressure after reperfusion therapy in patients with acute ischemic stroke

**DOI:** 10.1038/s41598-019-42240-8

**Published:** 2019-04-05

**Authors:** Kang-Ho Choi, Jae-Myung Kim, Ja-Hae Kim, Joon-Tae Kim, Man-Seok Park, Seong-Min Choi, Seung-Han Lee, Byeong C. Kim, Myeong-Kyu Kim, Ki-Hyun Cho

**Affiliations:** 1 Department of Neurology, Chonnam National University Hospital, Gwangju, Korea; 2 Department of Neurology, Chonnam National University Hwasun Hospital, Hwasun, Korea; 3 Department of Nuclear Medicine, Chonnam National University Hospital, Gwangju, Korea; 4 Molecular Imaging Center, Chonnam National University Hwasun Hospital, Hwasun, Korea

## Abstract

We investigated the relationship between the mean blood pressure (BP) at 24–72 h and the clinical outcomes after acute ischemic stroke (AIS) in patients treated with reperfusion therapy. The primary outcome was measured using the modified Rankin Scale (mRS) at 3 months after AIS, and was based on the mean systolic BP at 24–72 h post-AIS. Favorable outcome was defined as mRS scores of 0–2. A total of 1,540 patients treated with reperfusion therapy were enrolled in the study. Favorable outcomes occurred more frequently in patients with BP ≤ 130/80 mmHg, and the risks of symptomatic intracranial hemorrhage and early neurological deterioration were lower in this optimal BP group. Multivariable analysis showed a significant association between mean BP ≤ 130/80 mmHg at 24–72 h and favorable outcomes at 3 months after AIS (odds ratio 2.95, 95% confidence interval 2.32–3.77, *p* < 0.001). Prespecified subgroup analyses showed that BP ≤ 130/80 mmHg had a more significant impact on clinical outcome in patients with recanalization than in those without recanalization. These data indicate that a mean BP of ≤ 130/80 mmHg at 24–72 h post-AIS is independently associated with favorable outcomes in patients treated with reperfusion therapy, particularly in those with recanalization.

## Introduction

Blood pressure (BP) control is one of the most important factors affecting clinical outcomes in patients with acute ischemic stroke (AIS). There are two opposing concerns about the optimization of BP during the acute phase of AIS. Low BP may lead to hypoperfusion of ischemic tissue, particularly in the setting of persistent vessel occlusion, and may also have an adverse effect on collateral flow to the penumbrae^1,2^. By contrast, high BP contributes to reperfusion injury of ischemic tissue, particularly in the setting of recanalization of the occluded vessel after reperfusion therapy. High BP around the time of reperfusion therapy carries an increased risk of intracerebral hemorrhage, edema formation, and endothelial dysfunction^3,4^. Therefore, it is important to identify an optimal target BP and it may be beneficial to maintain low BP during the acute phase of AIS in patients treated with reperfusion therapy, especially in those with recanalization.

The recent American College of Cardiology/American Heart Association (ACC/AHA) task force guidelines for the management of high BP recommend more aggressive thresholds and goals for treatment than those outlined in the previous guidelines (BP ≤ 130/80 mmHg rather than ≤ 140/90 mmHg) in patients with AIS > 72 h after symptom onset^5^. However, there are no recommendations or previous reports concerning the optimal target BP at 24–72 h after AIS in patients treated with reperfusion therapy^5,6^. Furthermore, the effect of BP during this period post-AIS on functional outcomes in patients with complete recanalization after reperfusion therapy, which may directly influence reperfusion injury, is mostly unknown^7^.

The primary objective of this study was to identify an optimal BP by studying the relationship between BP at 24–72 h after AIS and the clinical outcomes in patients treated with reperfusion therapy. The study primarily focused on the subgroup of patients with complete recanalization after reperfusion therapy.

## Methods

### Subjects

This single-center study evaluated data from a prospective web-based acute stroke registry^8^. Patients admitted to a comprehensive stroke center between January 2007 and December 2016 were consecutively enrolled if they (1) had AIS and were seen within 6 h of symptom onset; (2) were treated with reperfusion therapy, including intravenous (IV) tissue plasminogen activator and/or mechanical thrombectomy (MT); (3) had acute ischemic lesions on brain imaging; and (4) underwent regular BP measurements during the initial 72 h after AIS. Patients with an uncontrolled underlying medical disease, such as an end-stage malignant tumor, severe renal disease (estimated glomerular filtration rate ≤ 15 mL·min^−1^·1.73 m^−2^), or liver disease (Child-Pugh class B or higher), were excluded.

Clinical and laboratory data were collected from all patients. Underlying stroke risk factors, including hypertension (prior use of antihypertensive medication), diabetes mellitus (prior use of diabetes medication or glycated hemoglobin >6.5% at admission), dyslipidemia (prior use of lipid-lowering medication), coronary artery disease, and current smoking, were assessed. The study protocol was approved by the institutional review board of Chonnam National University Hospital. All clinical investigations were conducted in accordance with the principles of the Declaration of Helsinki. Written informed consent was obtained from each patient or a family member.

### Clinical assessment and outcome measurements

The severity of neurological deficit was assessed during admission by using the National Institutes of Health Stroke Scale (NIHSS) score^9^. Clinical information on the outcome after discharge was obtained prospectively during routine clinic visits or via telephone interviews with patients or their caregivers at 3 months after the qualifying event. BP measurements were obtained every 2 h until 72 h post-AIS. The patients underwent magnetic resonance imaging (MRI) on admission and at 1 week after admission.

In patients treated with MT using a Solitaire (Covidien, Irvine, CA, USA) or Trevo (Stryker, Kalamazoo, MI, USA) device within 6 h of symptom onset, complete recanalization was defined as a Thrombolysis in Cerebral Infarction score of IIb or higher at the end of the endovascular procedure^10^. Patients eligible for MT were those with an occlusion of the carotid artery, middle cerebral artery (M1 or M2), anterior cerebral artery (A1), posterior cerebral artery (P1), or basilar artery, without restriction on age or neurological severity. In patients treated with IV thrombolysis only, arterial recanalization was assessed with computed tomography (CT) angiography at 24 h post-stroke. CT or MRI was performed if the symptoms worsened. Clinical and neuroimaging findings were used to group patients into five stroke subtypes according to the Trial of Org 10172 in Acute Stroke Treatment classification: (1) large-artery atherosclerosis, (2) small-vessel occlusion, (3) cardioembolism, (4) stroke of other determined etiology, and (5) stroke of undetermined etiology^11^.

The primary outcome was assessed using the modified Rankin Scale (mRS) at 3 months after the onset of symptoms. A favorable outcome (functional independence) was defined as an mRS score of 0–2^12^. The secondary outcomes (defined below) in the period >24 h after symptom onset were also evaluated. Early clinical improvement (ECI) was defined as an improvement of ≥ 4 points on the NIHSS, and early neurological deterioration (END) was defined as an aggravation by ≥ 1 point in motor power, or a worsening by ≥ 2 points in the total NIHSS score within 7 days of symptom onset^13,14^. Hemorrhagic transformation (HT) was considered to be present when the follow-up gradient echo MRI or CT scan revealed findings consistent with newly developed blood extravasation. Symptomatic intracranial hemorrhage (sICH) was defined as extravascular blood in the brain or cranium confirmed by neuroimaging and associated with neurological deterioration of ≥ 4 points on the NIHSS^15^. The MRI or CT scans were independently reviewed by two blinded investigators to determine the presence of HT or intracranial hemorrhage.

Appropriate treatment was provided to all patients according to best practice guidelines established by the AHA and ASA^6,16,17^. The BP goals after reperfusion therapy and the use of specific antihypertensive drug classes were determined by the physician’s preference and the clinical status of the patient. The standard of care at our government-initiated comprehensive tertiary stroke center was to maintain a BP of < 180/105 mmHg for several days in patients treated with reperfusion therapy in accordance with the AHA recommendations for the management of AIS^16^. The BP goals were lowered at 0–72 h post-reperfusion therapy (particularly if successful recanalization was achieved or if sICH occurred after reperfusion therapy) in accordance with the preference of the treating physician and previous reports about higher BP levels and an increased likelihood of poor outcomes^4,18,19^.

### Statistical analysis

Differences between the groups were analyzed using Student’s *t-*tests or the Kruskal-Wallis test for continuous variables. The chi-square test or Fisher’s exact test was used for categorical variables. Values of *p* < 0.05 were considered statistically significant. The Cochran-Mantel-Haenszel (CMH) test for shift analysis and dichotomized analysis was used to analyze the mRS score distribution^20^.

A backward stepwise logistic regression analysis was performed to assess the odds ratios (ORs) and the corresponding 95% confidence intervals (CIs) for the primary outcome. Adjustments were performed for the following variables, according to their clinical significance and the results of prior studies: age, sex, diabetes mellitus, dyslipidemia, atrial fibrillation, smoking, NIHSS score at 24 h, recanalization, and MT. Variables that were not significant (*p* > 0.2) were sequentially removed from the final model. A 2-sided *p*-value of < 0.05 was established as the level of significance.

A prespecified subgroup analysis for heterogeneity in BP effect was performed, with subgroups defined according to age (<70 years or ≥70 years), sex, baseline NIHSS score (≤12 or >12), type of reperfusion therapy (IV thrombolysis only or MT), large-vessel occlusion status (yes or no), and recanalization status (complete recanalization or no recanalization). Optimal BP was assessed through dichotomization based on threshold estimations of BP as a continuous variable for the main analysis. Sensitivity analysis was performed to assess which BP range was optimal. In the sensitivity analysis, the optimal BP was examined using the following categorization: ≤110 mmHg, >110 to ≤120 mmHg, >120 to ≤130 mmHg, >130 to ≤140 mmHg, >140 to ≤150 mmHg, and >150 mmHg. Statistical analyses were performed using SAS v. 9.4 (SAS Institute Inc., Cary, NC, USA) and R 3.3.1 (R Foundation for Statistical Computing, Vienna, Austria).

## Results

### Patient characteristics

The study included 1,540 patients with AIS treated with reperfusion therapy. To evaluate the relationship between BP and functional outcome, we analyzed the correlation between the mean systolic BP (SBP) at 24–72 h and the mRS score at 3 months after AIS; a statistically significant linear association was seen between the mean SBP and the mRS score (eFig. 1A Supplementary Material). Receiver operating characteristic curves were generated to determine the optimal BP in patients treated with reperfusion therapy; the following BP values were selected using multiple threshold estimations: SBP ≤ 130 mmHg and diastolic BP (DBP) ≤ 80 mmHg (eFigure 1B Supplementary Material). The optimal BP group was defined as patients with a mean SBP of ≤ 130 mmHg and a DBP of ≤ 80 mmHg; the hypertensive BP group was defined as those with an SBP of > 130 mmHg or a DBP of > 80 mmHg.

The clinical and biochemical characteristics of the two BP groups are shown in Table 1. No significant differences in the history of vascular risk factors were seen between the two groups, except for hypertension; no differences in stroke subtypes were seen. The mean age, proportion of female patients, and NIHSS scores on admission and at 24 h were higher in the hypertensive group than in the optimal BP group. The hypertensive group also had poorer risk factor control, indicated by increased total and low-density lipoprotein cholesterol levels and fasting blood glucose level. This group also had poorer BP control than the optimal BP group. The percentage of patients who underwent MT and achieved complete recanalization was lower in the hypertensive group than in the optimal BP group.

**Table 1:** Clinical and biochemical characteristics of the optimal and hypertensive blood pressure groups.

	Hypertensive BP > 130/80 mmHg (n = 815)	Optimal BP ≤ 130/80 mmHg (n = 725)	*p-*Value^*^
Age in years, mean ± SD	70.5 ± 11.2	67.7 ± 12.5	<0.001
Male sex, n (%)	428 (52.5)	431 (59.4)	0.007
**Risk factors, n (%)**			
Hypertension	653 (80.1)	387 (53.4)	<0.001
Diabetes mellitus	249 (30.6)	194 (26.8)	0.113
Atrial fibrillation	338 (41.5)	293 (40.4)	0.712
Dyslipidemia	120 (14.7)	98 (13.5)	0.545
Smoking	251 (30.8)	258 (35.6)	0.052
**Biochemical variables, mean ± SD**			
Total-C, mg/dL	180.9 ± 41.1	175.9 ± 41.8	0.017
LDL-C, mg/dL	116.8 ± 34.7	112.6 ± 33.7	0.017
Triglyceride, mg/dL	101.3 ± 59.5	97.7 ± 58.1	0.241
HDL-C, mg/dL	47.2 ± 13.4	46.3 ± 16.6	0.226
FBS, mg/dL	131.9 ± 48.3	122.1 ± 38.3	<0.001
**TOAST classification, n (%)**			0.063
LAA	283 (34.7)	228 (31.4)	
SVO	35 (4.3)	20 (2.8)	
CE	348 (42.7)	307 (42.3)	
OE	4 (0.5)	6 (0.8)	
UD	145 (17.8)	164 (22.6)	
Initial NIHSS score, mean ± SD	11.3 ± 5.1	10.6 ± 5.0	0.010
BP first 24 h after AIS, mean ± SD			
Systolic BP, mmHg	150.0 ± 23.2	129.4 ± 20.5	<0.001
Diastolic BP, mmHg	90.3 ± 13.6	80.0 ± 13.2	<0.001
NIHSS score at 24 h, mean ± SD	9.7 ± 6.9	7.1 ± 6.2	<0.001
BP 24–72 h after AIS, mean ± SD			
Systolic BP, mmHg	140.2 ± 8.4	119.4 ± 8.1	<0.001
Diastolic BP, mmHg	87.9 ± 9.5	76.2 ± 5.8	<0.001
**Reperfusion therapy**, **n (%)**			
IV thrombolysis	689 (84.5)	629 (86.8)	0.244
Mechanical thrombectomy	258 (31.7)	279 (38.5)	0.006
Large-artery occlusion	567 (69.6)	516 (71.2)	0.672
Recanalization	299 (52.7)	300 (58.1)	0.030

^*^Continuous variables were compared between groups using Student’s *t*-tests, one-way analyses of variance, or Mann-Whitney tests. The chi-square test was used for non-continuous variables.

BP, blood pressure; AIS, acute ischemic stroke; SD, standard deviation; Total-C, total cholesterol; LDL-C, low-density lipoprotein cholesterol; HDL-C, high-density lipoprotein cholesterol; FBS, fasting blood sugar; TOAST, Trial of Org 10172 in Acute Stroke Treatment; LAA, large-artery atherosclerosis; SVO, small-vessel occlusion; CE, cardioembolism; OE, stroke of other determined etiology; UD, stroke of undetermined etiology; NIHSS, National Institutes of Health Stroke Scale; mRS, modified Rankin Scale; IV, intravenous.

### Clinical outcomes

**Figure 1:**
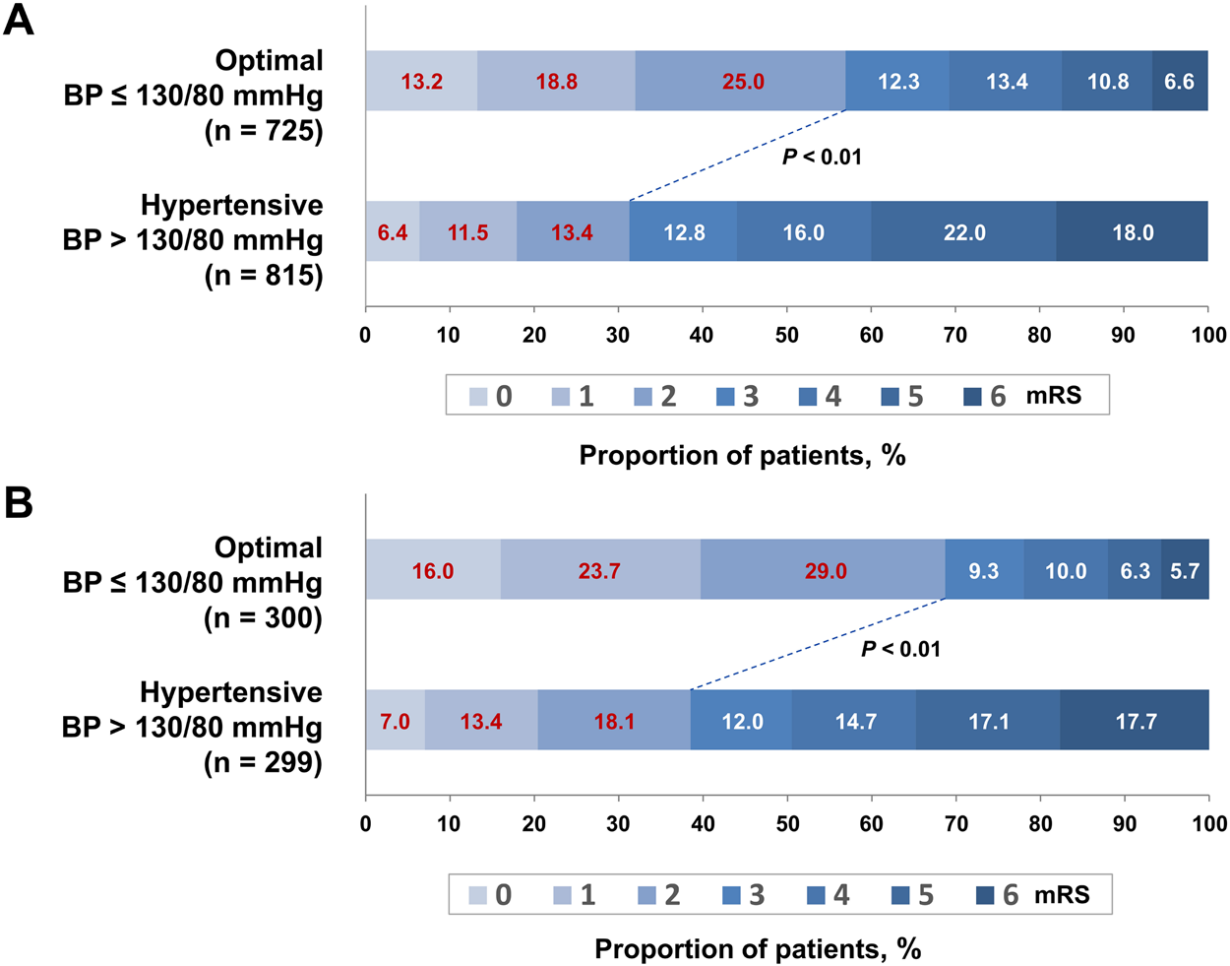
Primary outcome according to blood pressure (BP). Distribution of the modified Rankin Scale (mRS) scores among all patients (**A**) and in patients with recanalization (**B**) classified according to BP at 3 months after ischemic stroke. The lines indicate differences in mRS categories (mRS scores 0–2 vs. 3–6) between groups according to BP. The *p*-value refers to the significance level of the chi-square test used to analyze the proportions of mRS scores.

The primary outcome of functional independence was confirmed in 668 (43.4%) patients, with 413 (57.0%) in the optimal BP group and 255 (31.3%) in the hypertensive group (*p* < 0.01 in the chi-square test, Fig. 1A and Table 2). The CMH test for shift analysis also revealed a statistically significant shift in the distribution of mRS scores at 3 months after the onset of AIS, favoring the optimal BP group. This finding was consistent with that of the dichotomized analysis (*p* < 0.01).

**Table 2:** Association between mean BP ≤ 130/80 mmHg at 24–72 h after acute ischemic stroke and clinical outcomes.

	Optimal BP ≤ 130/80 mmHg	Hypertensive BP > 130/80 mmHg	Absolute difference*	Adjusted OR (95% CI)^†^
***All patients***	**n = 725**	**n = 815**		
**Primary outcome, n (%)**				
Functional independence at 3 months	413 (57.0)	255 (31.3)	25.7	2.95 (2.32–3.77)
**Secondary outcomes, n (%)**				
Early clinical improvement	164 (22.6)	132 (16.2)	6.4	1.15 (0.90–1.47)
Early neurological deterioration	99 (13.7)	214 (26.3)	12.6	0.65 (0.47–0.89)
Hemorrhagic transformation	161 (22.2)	261 (32.0)	9.8	0.67 (0.52–0.86)
Symptomatic intracranial hemorrhage	18 (2.5)	53 (6.5)	4.0	0.44 (0.24–0.76)
***Patients with recanalization***	**n = 300**	**n = 299**		
**Primary outcome, n (%)**				
Functional independence at 3 months	206 (68.7)	115 (38.5)	30.2	3.56 (2.64–4.81)
**Secondary outcomes, n (%)**				
Early clinical improvement	107 (35.7)	72 (24.1)	11.6	1.35 (0.87–2.11)
Early neurological deterioration	24 (8.0)	61 (20.4)	12.4	0.47 (0.24–0.91)
Hemorrhagic transformation	60 (20.0)	137 (45.8)	25.8	0.32 (0.21–0.47)
Symptomatic intracranial hemorrhage	2 (0.7)	28 (9.4)	8.7	0.08 (0.02–0.27)

BP, blood pressure; CI, confidence interval; OR, odds ratio.

^*^Absolute differences are reported in percentage points.

^**†**^Adjusted variables: age, sex, diabetes mellitus, dyslipidemia, atrial fibrillation, smoking, National Institutes of Health Stroke Scale score at 24 h, recanalization, and mechanical thrombectomy.

Complete recanalization was achieved in 599 of the 1,083 (55.3%) patients with large-artery occlusion; of these, 300 (58.1%) were in the optimal BP group and 299 (52.7%) were in the hypertensive group (*p* = 0.03, Table 1). Patients with recanalization had a higher rate of functional independence than those without recanalization, including patients with partial recanalization (53.6% vs. 19.8%, *p* < 0.001). Among patients with complete reperfusion, the number of those with a favorable outcome was significantly higher in the optimal BP group than in the hypertensive group (68.7% vs. 38.5%, *p* < 0.001 in the chi-square test and shift analysis with the CMH test; Fig. 1B and Table 2). The 3-month mortality rate was higher in the hypertensive group (17.7%) than in the optimal BP group (5.7%, *p* < 0.001; Fig. 1B).

With respect to secondary outcomes, 296 patients (19.2%) had ECI, which was more prevalent in the optimal BP group than in the hypertensive group (22.6% vs. 16.2%, *p* < 0.001; Table 2). END occurred in 313 patients (20.3%), and was significantly less prevalent in the optimal BP group than in the hypertensive group (13.7% vs. 26.3%, *p* < 0.001; Table 2). HT was more frequent in the hypertensive group than in the optimal BP group (32.0% vs. 22.2%, *p* < 0.001). The frequency of sICH was also significantly higher in the hypertensive group than in the optimal BP group (6.5% vs. 2.5%, *p* < 0.001; Table 2). The difference in the prevalence of HT and sICH between the two BP groups was notably higher in patients with recanalization (Table 2).

In the final multivariable analysis, which included adjustments for confounding factors, a BP of ≤ 130/80 mmHg at 24–72 h post-AIS was significantly associated with a favorable outcome at 3 months post-AIS (OR 2.95, 95% CI 2.32–3.77, *p* < 0.001; Table 2). The optimal BP of ≤ 130/80 mmHg was also significantly associated with END (OR 0.65, 95% CI 0.47–0.89, *p* = 0.008), HT (OR 0.67, 95% CI 0.52–0.86, *p* = 0.002), and sICH (OR 0.44, 95% CI 0.24–0.76, *p* = 0.005; Table 2). In patients with recanalization, the relationship between BP ≤ 130/80 mmHg and functional outcome at 3 months after AIS was independently associated with significant values (OR 3.56, 95% CI 2.64–4.81, *p* < 0.001; Table 2).

**Figure 2:**
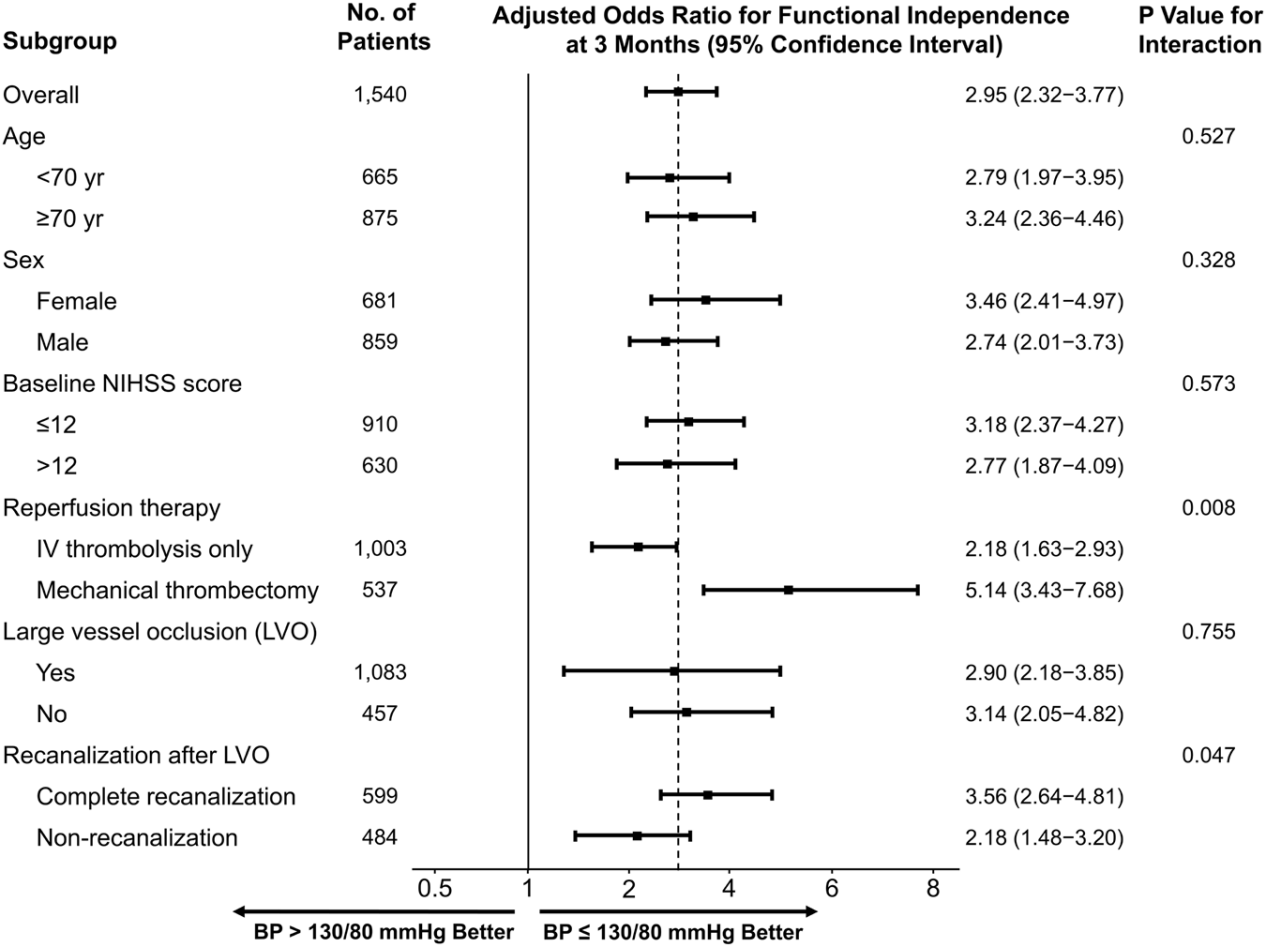
Subgroup analyses of the primary outcome. The forest plot shows that the difference in the odds ratio for functional independence (defined as a modified Rankin Scale score of 0–2) at 3 months favored the blood pressure ≤ 130/80 mmHg group across all prespecified subgroups. NIHSS, National Institutes of Health Stroke Scale; IV, intravenous. Adjusted variables: age, sex, diabetes mellitus, dyslipidemia, atrial fibrillation, smoking, NIHSS score at 24 h, recanalization, and mechanical thrombectomy.

In prespecified subgroup analyses, there was a significant heterogeneity in the ORs for favorable outcomes associated with the BP group in patients with and without recanalization and in those treated with MT and IV thrombolysis only (Fig. 2). BP ≤ 130/80 mmHg was associated with significantly higher odds of functional independence at 3 months after AIS in the recanalized group when compared with non-recanalized patients (*p* = 0.047). Optimal BP at 24–72 h post-AIS also had a more significant impact on clinical outcome in patients treated with MT than in those treated with IV thrombolysis only (*p* = 0.008).

**Figure 3:**
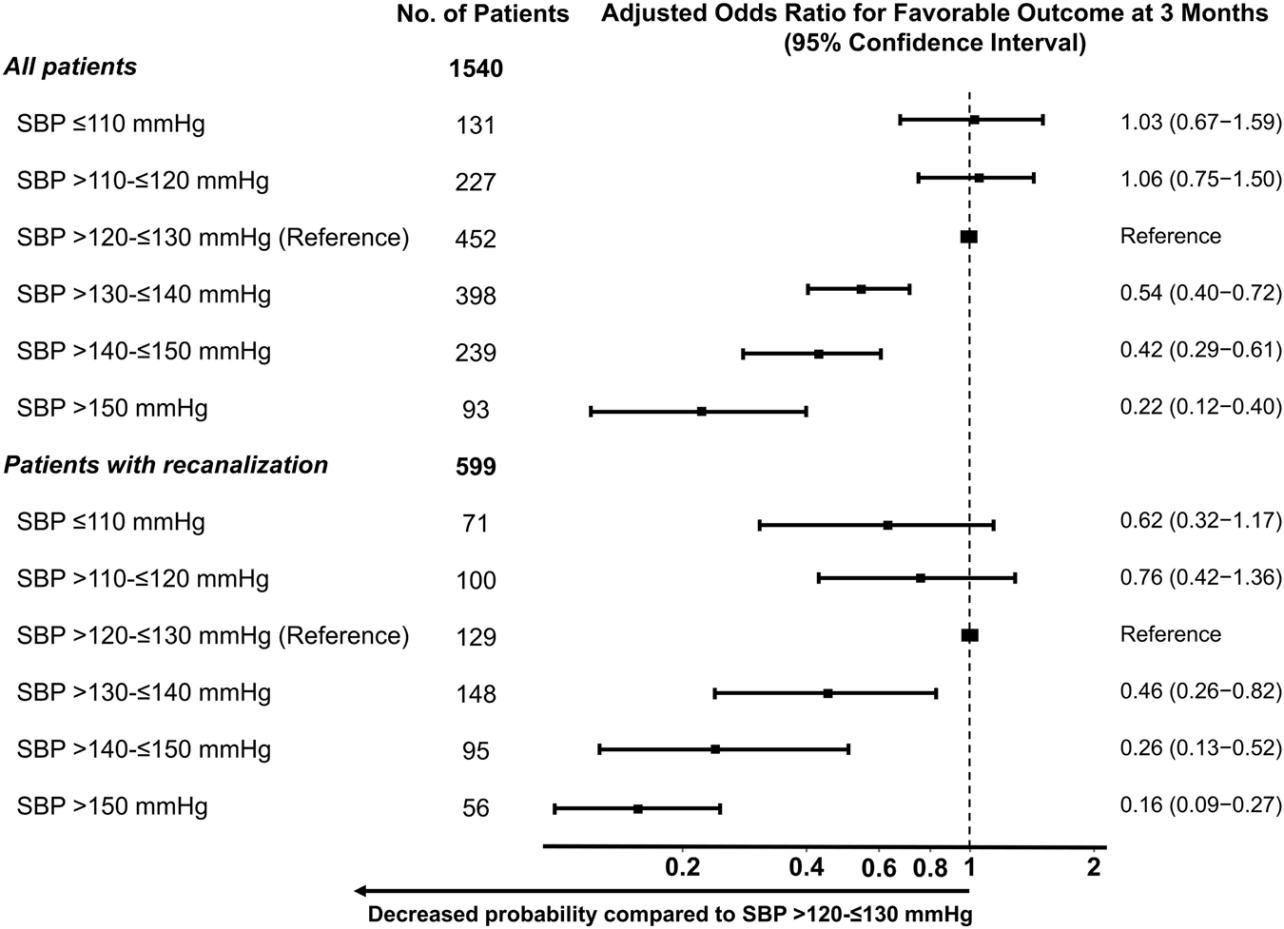
Association of different systolic blood pressure levels with a 3-month favorable outcome after reperfusion therapy in all patients and in patients with recanalization. *p < 0.01. A multiple logistic regression test was used to analyze odds ratios. CI, confidence interval. Adjusted variables: age, sex, diabetes mellitus, dyslipidemia, atrial fibrillation, smoking, National Institutes of Health Stroke Scale score at 24 h, recanalization, and mechanical thrombectomy.

Sensitivity analysis showed a significant decrease in the probability of a favorable outcome in patients with SBP > 130 mmHg (vs. >120 to ≤130 mmHg), with the point estimate of the OR favoring the reference SBP (>120 to ≤130 mmHg) when compared with higher SBP (≤140 mmHg or ≤150 mmHg; Fig. 3). There was no evidence for a further effect of SBP lowering below 120 mmHg on a favorable outcome (Fig. 3).

## Discussion

This study demonstrates for the first time that maintaining a mean BP of ≤ 130/80 mmHg at 24–72 h after AIS is associated with higher odds of a favorable outcome at 3 months post-AIS and a lower likelihood of sICH and END than maintaining a mean BP of > 130/80 mmHg after adjustment for potential confounding factors in patients with AIS treated with reperfusion therapy. There was a linear relationship between the SBP level at 24–72 h and the mRS scores at 3 months after AIS in those patients. In addition, we found that BP ≤ 130/80 mmHg was associated with much higher odds of a favorable outcome at 3 months after AIS in patients with recanalization when compared with those without recanalization.

Our results extend beyond recently developed guidelines and previous reports that deal with only the first 24 h or >72 h after AIS and did not report on the control of BP during the intervening 24–72 h in patients with AIS treated with reperfusion therapy. In previous studies, maintenance of BP at levels lower than those achieved using conservative treatment during the first 24 h after symptom onset was associated with improved prognosis in patients with AIS treated with reperfusion therapy^18,19,21–24^. However, no results exist to guide optimal BP management at 24–72 h after reperfusion therapy. The current study revealed that low BP levels during the 24–72 h period post-AIS are associated with good short- and long-term outcomes, and BP ≤ 130/80 mmHg may be optimal in patients treated with reperfusion therapy.

These results thus lend further credence to our earlier suggestion that maintaining low BP during the acute phase could improve the clinical outcome in patients treated with reperfusion therapy. Our findings are in line with recent guidelines and research that suggest more intensive control of BP, proposing a lower BP target of ≤ 130/80 mmHg rather than ≤ 140/90 mmHg^5,25,26^. Given that guidelines recommend a lower BP target within the first 24 h in patients with AIS treated with reperfusion therapy (<180/105 mmHg) than in patients with AIS without reperfusion therapy (<220/120 mmHg), patients with AIS treated with reperfusion therapy may benefit from a lower BP target during the 24–72 h period post-AIS than that in patients with AIS without reperfusion therapy^5,6^. In patients with AIS without reperfusion therapy, a recent sub-study of a randomized controlled trial (RCT) provided evidence that BP reduction below 140/90 mmHg at 24–48 h after stroke onset (but not in the first 24 h) potentially reduces the incidence of death or major disability, as well as that of recurrent stroke, at 3 months after symptom onset^27^. Therefore, in patients treated with reperfusion therapy, BP ≤ 130/80 mmHg, which is lower than the ≤ 140/90 mmHg suggested for patients without reperfusion therapy in the above study, may be a reasonable optimal target during the 24–72 h period post-AIS, as shown in our study.

This may be particularly pertinent in patients with recanalization after reperfusion therapy, especially MT. This study shows that maintenance of the optimal BP level of ≤ 130/80 mmHg during the 24–72 h period post-AIS has a more significant impact on clinical outcome in patients with recanalization than in those without recanalization, and in those treated with MT than in those treated with IV thrombolysis only. MT is now the standard of care for patients with large-vessel occlusion ischemic stroke, and the recanalization success rate has significantly increased since the introduction of MT^28^. Because high BP may further increase the risk of reperfusion injury related to intracerebral hemorrhage, edema formation, and endothelial dysfunction in patients with recanalization after reperfusion therapy, it may be more beneficial and important to adjust the BP within the optimal level of ≤ 130/80 mmHg during the 24–72 h period post-AIS in patients with recanalization after MT^3,4^.

This study has definite limitations, and the data must be interpreted with caution. First, a cause-and-effect relationship between BP levels and the outcomes cannot be assumed from these data. As this is an observational study and not a randomly assigned trial, our data are subject to potential bias and confounding factors. Therefore, it is possible that BP is only a marker of prognosis rather than a cause. It is well known that there is a significant decrease in SBP level between the time of admission and 24 h after thrombolysis in patients with recanalization, but not in patients whose vessels remain occluded^29^. In this respect, BP ≤ 130/80 mmHg at 24–72 h post-AIS may not be the cause of a favorable outcome at 3 months post-AIS, but may be considered to simply reflect a successful recanalization. However, as the sub-analyses in only patients with recanalization revealed a more significant effect of lower BP on prognosis than that seen in patients without recanalization, BP ≤ 130/80 mmHg may be optimal in patients treated with reperfusion therapy. Our observations warrant further investigation as part of a large RCT. Second, we did not collect detailed information about BP-lowering treatment, such as the type, dose, or timing of medication. Third, we did not systematically measure BP levels between 72 h and 3 months after reperfusion therapy, and could not determine whether this factor had any effect on clinical outcomes in our patients.

## Conclusions

The results of the present study indicate that BP ≤ 130/80 mmHg at 24–72 h after AIS is independently associated with favorable short- and long-term clinical outcomes in patients treated with reperfusion therapy. This may be particularly pertinent in patients with recanalization after reperfusion therapy, especially MT. These findings have significant potential implications for acute stroke care, as they support the need to initiate treatment for high BP early, rather than conservatively delaying therapy to 72 h after symptom onset. Future large and well-designed prospective RCTs are required to confirm the optimal target BP at 24–72 h after AIS in patients treated with reperfusion therapy.

## Supplementary information

Supplementary figure
