# Supplementary material for: Optimal blood pressure after reperfusion therapy in patients with acute ischemic stroke
Source: Sci Rep. 2019 Apr 5;9:5681. doi: 10.1038/s41598-019-42240-8 (PMC6450931; doi:10.1038/s41598-019-42240-8)
Supplement: Supplementary file 1 — Supplementary figure [file 41598_2019_42240_MOESM1_ESM.pdf]

Optimal blood pressure after reperfusion therapy in patients with acute ischemic stroke

Kang-Ho Choi, MD, PhD[1,2*†], Jae-Myung Kim, MD[1†], Ja-Hae Kim, MD, PhD[3,4*], Joon-Tae Kim, MD, PhD[1], Man-Seok Park, MD, PhD[1], Seong-Min Choi, MD, PhD[1], Seung-Han Lee, MD, PhD[1], Byeong C. Kim, MD, PhD[1], Myeong-Kyu Kim, MD, PhD[1], Ki-Hyun Cho, MD, PhD[1]

[1]Department of Neurology, Chonnam National University Hospital, Gwangju, Korea

[2]Department of Neurology, Chonnam National University Hwasun Hospital, Hwasun, Korea

[3]Department of Nuclear Medicine, Chonnam National University Hospital, Gwangju, Korea

[4]Molecular Imaging Center, Chonnam National University Hwasun Hospital, Hwasun, Korea

***Authors for correspondence and requests for reprints:**

Kang-Ho Choi, MD, PhD

Department of Neurology, Chonnam National University Hwasun Hospital 322

Seoyang-Ro, Hwasun, Chonnam, Republic of Korea

Tel.: +82-62-220-6137, fax: +82-62-228-3461

E-mail: ckhchoikang@hanmail.net, ckhchoikang@chonnam.ac.kr

Ja-Hae Kim, MD, PhD

Department of Nuclear Medicine, Chonnam National University Hospital

8 Hakdong, Donggu, Gwagnju, Republic of Korea

Tel.: +82-62-220-6155

E-mail: jhbt0607@hanmail.net

[†]These authors contributed equally to this work.

Supplementary figure

eFigure 1. (A) Distribution of modified Rankin Scale scores according to systolic blood pressure. (B) Optimal cutoff values for systolic and diastolic blood pressures for favorable outcomes.

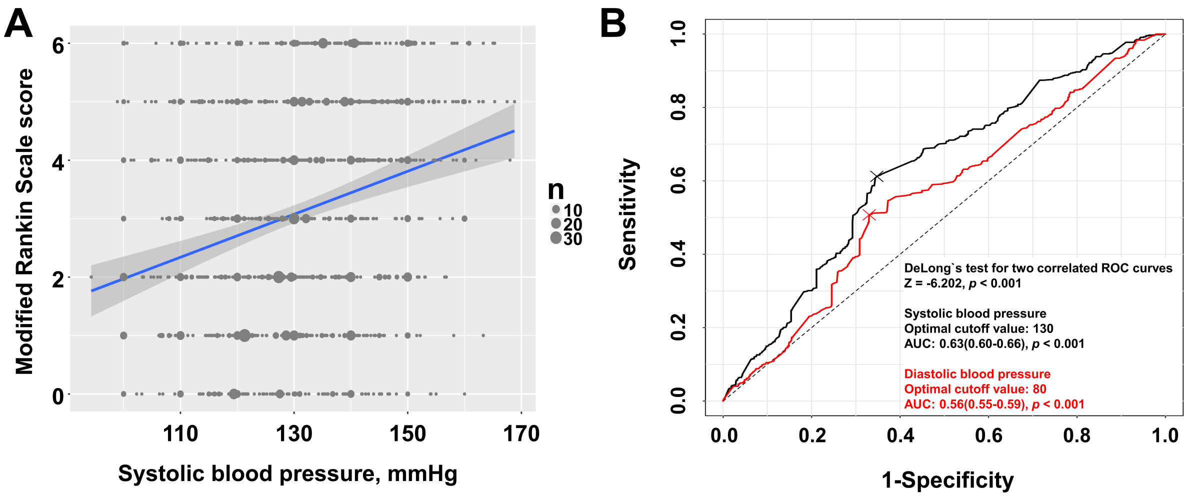

ROC, receiver operating characteristic; AUC, area under the curve.
